# Successful reversal of hepatic visceral crisis in HER2-positive metastatic breast cancer: a case report

**DOI:** 10.3389/fonc.2026.1758742

**Published:** 2026-05-22

**Authors:** Joan Manuel Moreno Luján, Jorge Luis Soriano Lorenzo, Miguel Martínez Rubiño, Sònia Servitja Tormo, Tamara Martos Cárdenas, María Martínez García, Joan Albanell Mestres

**Affiliations:** Hospital del Mar Research Institute Barcelona, Department of Medical Oncology, Hospital del Mar, Barcelona, Spain

**Keywords:** dual HER2 blockade, hepatic visceral crisis, HER2-positive breast cancer, metastatic breast cancer, pseudocirrhosis

## Abstract

**Introduction:**

Hepatic visceral crisis (HVC) in the setting of HER2-positive metastatic breast cancer (mBC-HER2+) is an infrequent but often catastrophic presentation. Management remains particularly challenging, largely because patients with marked hepatic dysfunction are routinely excluded from clinical trials, leaving clinicians with little prospective evidence to guide decisions. For this reason, observations from well-documented cases continue to offer practical insights when conventional pathways fall short.

**Case presentation:**

A 51-year-old woman with HER2-positive metastatic breast cancer (mBC) and widespread bilobar liver metastases, BRCA wild-type, presented with rapidly progressive jaundice and a marked decline in performance status. Initial laboratory tests revealed severe hepatic dysfunction—total bilirubin 31.4 mg/dL, AST 462 U/L, ALT 185 U/L, and LDH >1800 U/L—meeting criteria for hepatic visceral crisis (HVC). Given the clinical severity, inpatient treatment was promptly initiated with trastuzumab and pertuzumab every three weeks, combined with weekly paclitaxel at a 50% dose reduction. During this initial phase, the patient showed early clinical and biochemical improvement; however, paclitaxel was discontinued after the second dose due to grade 2 toxicities, while anti-HER2 therapy was maintained without interruption.

**Discussion:**

Over the following cycles, liver function normalized, CA15–3 levels fell from 893 to 39.5 U/mL, and imaging demonstrated a sustained partial response. Radiologic assessment also revealed changes consistent with pseudocirrhosis—hepatic contour remodeling, capsular retraction, and venous alterations—findings that, in this context, were interpreted as treatment-related rather than evidence of progression. The patient’s course suggests that, when HER2 dependence is strong, rapid and continuous HER2-targeted therapy may restore organ function even from a state of profound hepatic failure. The brief exposure to dose-reduced paclitaxel likely served only as an initial cytoreductive step while avoiding further hepatic strain.

**Conclusion:**

This case illustrates that HVC in mBC-HER2+ is not uniformly irreversible. In carefully selected patients, early and sustained HER2-directed therapy can lead to meaningful clinical recovery when options appear limited. Such cases help bridge the evidence gap for this high-risk group and may inform future therapeutic considerations.

## 1 Introduction

Breast cancer remains the most frequently diagnosed malignancy among women worldwide (1). Approximately 15–20% of cases overexpress or amplify the HER2 oncogene, a subtype associated with aggressive clinical behavior and a higher propensity for early visceral involvement (2). The identification of HER2 as a highly actionable therapeutic driver and the subsequent development of monoclonal antibodies, tyrosine kinase inhibitors, and antibody–drug conjugates have radically transformed outcomes in HER2-positive breast cancer, establishing it as one of the most successful paradigms in precision oncology (3).

Despite these advances, a subset of patients still experiences rapidly progressive disease driven by aggressive tumor biology, including visceral crisis (VC), a life-threatening state of acute organ dysfunction arising from uncontrolled metastatic progression. According to the ABC-5/ESO–ESMO guidelines, VC is defined by rapidly worsening organ function—such as fulminant hepatic failure or pulmonary lymphangitic carcinomatosis—requiring immediate initiation of therapies capable of achieving a rapid antitumor effect (4). Prognosis remains poor, with historical series reporting median overall survival of less than four months (5).

Evidence guiding the management of VC in HER2-positive disease is scarce, as patients with severe hepatic or pulmonary dysfunction are systematically excluded from pivotal clinical trials (5). Consequently, well-documented case reports play a critical role in describing therapeutic responses in high-risk scenarios that fall outside the scope of prospective evidence.

Here, we describe a patient with mBC-HER2+ who presented with hepatic visceral crisis and extreme hyperbilirubinemia, and who achieved complete biochemical recovery and sustained radiologic response following early dual HER2 blockade combined with dose-adjusted chemotherapy.

## 2 Case description

A 51-year-old Caucasian woman without relevant comorbidities and no history of tobacco or alcohol use, with a maternal family history notable for a grandmother with gynecologic malignancy at approximately 50 years of age and breast cancer at around 65 years, and confirmed BRCA wild-type status, was diagnosed in April 2016 with right-sided invasive ductal carcinoma, luminal B subtype, HER2-positive (cT1bN0M0). She received neoadjuvant chemotherapy with anthracyclines followed by weekly paclitaxel, after which she underwent breast-conserving surgery. Histopathologic evaluation demonstrated a pathological complete response (ypT0N0M0). Adjuvant treatment included radiotherapy, one year of trastuzumab, and tamoxifen.

In February 2020, a local recurrence was treated with radical mastectomy. Adjuvant systemic therapy was recommended but declined by the patient due to prior poor tolerance to chemotherapy; consequently, no anti-HER2–directed therapy was administered. By May 2023, a second locoregional recurrence appeared at the mastectomy scar. During the subsequent two years, the patient exclusively pursued non-validated alternative therapies (including ozone therapy and high-dose vitamin C).

In May 2025, she presented to the emergency department with progressive clinical deterioration, including generalized malaise, abdominal distension, mucocutaneous jaundice, unintentional weight loss, and abdominal pain. Her functional status was markedly impaired (ECOG 3). On physical examination, an ulcerated right thoracic wall lesion measuring approximately 8 × 5 cm was observed. The liver edge was palpable 10 cm below the costal margin, and ascites was clinically evident.

Laboratory evaluation revealed a severe cholestatic–cytolytic pattern, with total bilirubin 31.4 mg/dL, AST 462 U/L, ALT 185 U/L, alkaline phosphatase 542 U/L, GGT 723 U/L, and LDH 1801 U/L (ULN < 250), consistent with acute hepatic failure. Tumor marker levels were markedly elevated, with CA 15–3 of 893 U/mL. Abdominal ultrasound demonstrated multiple bilobar solid hepatic lesions with intrahepatic biliary dilation in the absence of mechanical obstruction. Whole-body PET/CT confirmed extensive metastatic involvement of the liver, lungs, bone, and supra- and infradiaphragmatic lymph nodes (**Figure 1**).

**Figure 1:**
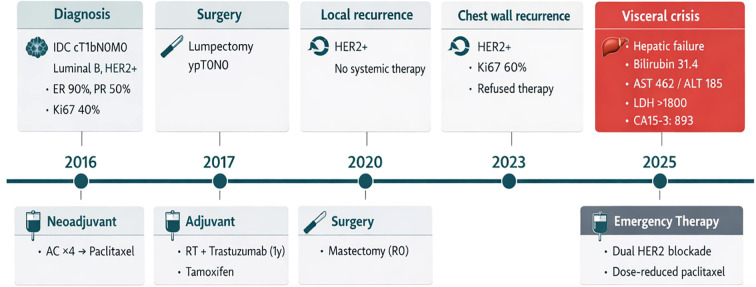
Chronological overview of the patient’s clinical course and treatment timeline.

The diagnostic assessment was challenged by the presence of extreme hyperbilirubinemia, requiring differentiation between malignant infiltration, biliary obstruction, drug-induced liver injury, and acute hepatitis. Mechanical obstruction was effectively excluded based on imaging findings, while the absence of recent hepatotoxic exposures and the clinical context made drug-induced liver injury unlikely. Acute hepatitis was considered less probable given the radiologic findings and the marked tumor burden. The combination of diffuse hepatic infiltration, rapid clinical deterioration, and absence of obstructive pathology supported the diagnosis of hepatic failure secondary to tumor infiltration.

A differential diagnosis including malignant biliary obstruction, acute hepatic failure of alternative etiologies, and diffuse metastatic progression was systematically considered. Ultimately, the integration of clinical, biochemical, and imaging findings led to the diagnosis of hepatic visceral crisis (HVC) secondary to rapidly progressive metastatic disease.

From a prognostic standpoint, the patient presented with multiple high-risk features, including severe hyperbilirubinemia (>30 mg/dL), markedly elevated LDH, poor performance status (ECOG 3), and extensive hepatic tumor burden, all of which are associated with an extremely poor expected outcome. Despite these adverse prognostic indicators, early initiation of biology-driven systemic therapy with pertuzumab, trastuzumab and adjusted dose of paclitaxel resulted in rapid clinical and biochemical improvement, further supporting the initial diagnostic interpretation.

Follow-up PET/CT after six cycles showed a marked metabolic response, with decreased uptake at the surgical scar, resolution of pulmonary lesions, disappearance of mediastinal and hilar lymphadenopathy, and resolution of multiple hypermetabolic hepatic foci, with post-treatment fibrotic remodeling (**Figure 2**; **Table 1**).

**Table 1:** Tumor response was assessed per RECIST 1.1 using CT morphology, with PET as supportive data.

Category	Site	Baseline (26 May 2025)	Follow-up (PET/CT 18 Sep 2025)	RECIST 1.1
Target lesion	Liver (Seg. VIII)	Multiple bilobar FDG-avid lesions	Single residual lesion 48 × 27 mm, hypodense, low FDG uptake (SUVmax 4.3)	PR
Non-target	Hilar/mediastinal nodes	FDG-avid lymphadenopathy	Complete resolution	CR
Non-target	Lung	Multiple metastatic nodules	Complete resolution	CR
Non-target	Bone (T9, pelvis, femur)	Multiple mixed FDG-avid lesions	Marked decrease; minimal residual uptake	Non-PD (residual disease)

Follow-up showed marked tumor reduction, with residual hepatic disease consistent with partial response. Nodal and pulmonary lesions achieved complete response, while bone disease remained non-progressive. No new lesions were identified; overall best response was partial response (PR).

**Figure 2:**
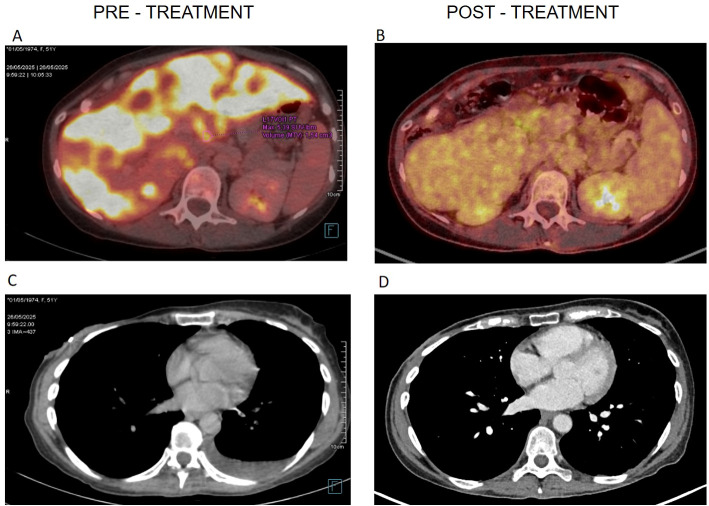
Comparative PET/CT imaging before and after treatment. **(A, C)** Baseline (pre-treatment): **(A)** Diffuse bilobar hepatic metastases with high metabolic activity. **(C)** Pleural effusion and costal metastatic lesion. **(B, D)** Post-treatment: **(B)** Near-complete metabolic resolution of hepatic lesions. **(D)** Resolution of pleural effusion and costal metastasis. Findings are consistent with a marked systemic response to therapy.

Biochemically, the patient experienced a sustained response (**Figure 3**; **Table 2**), with total bilirubin decreasing from 31.4 mg/dL at presentation to 1.1 mg/dL and CA 15–3 decreasing from 893 U/mL to 31.8 U/mL. Clinically, she showed full resolution of jaundice, reduction of hepatomegaly, and improvement in performance status from ECOG 3 at diagnosis to ECOG 1.

**Table 2:** Key laboratory parameters at presentation and during treatment.

Parameter	At diagnosis	Best post-treatments value	Normal range
Total bilirubin	31.4 mg/dL	1.1 mg/dL	≤ 1.2 mg/dL
AST	462 U/L	51 U/L	≤ 35 U/L
ALT	185 U/L	38 U/L	≤ 35 U/L
LDH	1801 U/L	306 U/L	≤ 250 U/L
CA15-3	893 U/mL	31.8 U/mL	≤ 35 U/mL

Summary of critical hepatic and tumor-related laboratory values, including total bilirubin, AST, ALT, LDH, and CA 15-3, reported at baseline and at nadir following initiation of therapy. Values demonstrate severe hepatic dysfunction at presentation and significant normalization after treatment.

**Figure 3:**
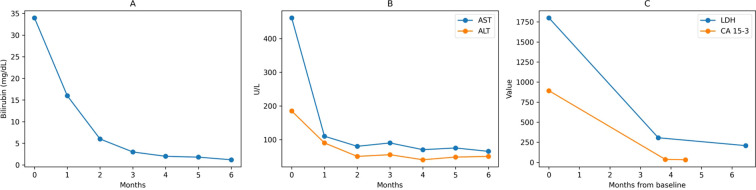
Biochemical evolution following treatment initiation. **(A)** rapid decline in total bilirubin levels. **(B)** improvement in transaminases (AST, ALT). **(C)** decrease in LDH and CA 15–3 levels, reflecting reduction in tumor burden.

At last follow-up in March 2026, the patient remained in very good general condition, with normalization of bilirubin levels and improvement in performance status to ECOG 1, while continuing maintenance trastuzumab and pertuzumab, with no evidence of disease progression.

## 3 Discussion

Visceral crisis (VC) in metastatic breast cancer represents one of the most severe oncologic emergencies, defined by rapidly progressive organ dysfunction attributable to uncontrolled tumor burden. Among its subtypes, hepatic visceral crisis (HVC)—characterized in ABC-5/ESO–ESMO guidelines by a rapid rise in bilirubin >1.5 × ULN in the absence of obstruction or Gilbert’s syndrome—is associated with the worst outcomes (4). Real-world cohorts consistently report median overall survival between 4.7 weeks and 11 months despite aggressive treatment (5), reflecting its exceptionally poor prognosis.

The present case stands out because a complete and sustained reversal of HVC was achieved despite a biochemical profile far more severe than typically documented. Most published cases describe bilirubin values of 2–12 mg/dL at presentation (5–7), whereas our patient presented with bilirubin 31.4 mg/dL, AST 462 U/L, ALT 185 U/L, and LDH >1800 U/L—findings compatible with fulminant hepatic failure. Recovery from such profound dysfunction is rarely observed, underscoring the clinical relevance of this outcome.

Current guidelines, including NCCN and ABC-5, recommend urgent systemic therapy with regimens capable of inducing rapid tumor shrinkage and restoring organ function (4, 8). Historically, platinum-based or multi-agent cytotoxic regimens have been favored in HVC because their clearance is less dependent on hepatic metabolism. However, retrospective series show limited benefit: platinum-based combinations achieve a median survival around 3.7 months, and heavily pretreated patients may survive only 4.7 weeks (6, 9). These results highlight the limitations of cytotoxic-centered strategies in this setting and the importance of treatment approaches that address the underlying tumor biology.

HER2-positive metastatic breast cancer is driven by a well-defined oncogenic pathway, offering a unique therapeutic opportunity during VC. Nevertheless, the optimal management of HER2-positive HVC remains uncertain. Pivotal trials such as CLEOPATRA and PERUSE excluded patients with bilirubin >1.5–2 mg/dL (10, 11), leaving a substantial evidence gap. Case reports and small series suggest that dual HER2 blockade with trastuzumab and pertuzumab—often combined with dose-adapted taxanes—can be effective despite significant organ dysfunction. Yang et al. reported a median survival of 11 months for patients treated with trastuzumab–pertuzumab plus chemotherapy (7), and other isolated reports describe durable responses in selected individuals (12).

This uncertainty contrasts with the HR-+/HER2- setting, where more robust evidence is available. In the RIGHT Choice trial, CDK4/6 inhibition plus endocrine therapy outperformed chemotherapy even in patients with high tumor burden or visceral crisis features, improving progression-free survival and delaying treatment failure (17). Taken together, these observations point to a broader idea: therapies targeting the underlying molecular driver can outperform purely cytotoxic approaches in the context of visceral crisis. This further supports prioritizing pathway-specific interventions in HER2-dependent disease whenever the clinical situation allows. However, unlike HR+/HER2– tumors, no controlled studies have evaluated anti-HER2 therapy versus chemotherapy during HVC, leaving clinicians reliant on case-based experience.

In our patient, early initiation and uninterrupted continuation of full-dose trastuzumab and pertuzumab appeared crucial. Mechanistically, trastuzumab (domain IV) and pertuzumab (domain II) prevent HER2 homo- and heterodimerization, inhibiting PI3K/AKT and MAPK signaling and generating rapid, synergistic antitumor activity (13). Despite marked hepatic dysfunction, maintaining targeted therapy was feasible and likely the primary driver of organ recovery.

Paclitaxel was administered at 50% of the standard weekly dose as a cautious cytoreductive bridge. Evidence supports dose-reduction strategies in patients with hepatic impairment because even mild dysfunction increases the risk of grade 3–4 hematologic toxicity (14). In this case, discontinuation of paclitaxel after two doses did not impede overall recovery, suggesting that sustained improvement derived predominantly from uninterrupted HER2 blockade rather than prolonged cytotoxic exposure.

Radiologic follow-up revealed features characteristic of pseudocirrhosis—hepatic atrophy, irregular contour, capsular retraction, and venous remodeling. Pseudocirrhosis is a relatively frequent phenomenon in patients with breast cancer and liver metastases, with reported incidences ranging from approximately 50% to 75% in radiologic series, particularly in the setting of high tumor burden and active systemic therapy. Large retrospective series report signs of portal hypertension in 38–81% of cases and clinically significant hepatic decompensation in approximately 10–12%, with median survival after diagnosis around 7.6 months; outcomes appear somewhat more favorable in HER2-positive disease (15, 16). In this case, the patient presented with extensive bilobar hepatic involvement, representing a high-risk scenario for pseudocirrhosis, further compounded by the rapid and marked tumor response achieved with HER2-targeted therapy, a mechanism known to promote abrupt parenchymal remodeling and capsular retraction. Proposed pathophysiological mechanisms include rapid tumor shrinkage with capsular scarring, desmoplastic response, or nodular regenerative hyperplasia (15). Given its heterogeneous nature, pseudocirrhosis should not be assumed to be benign or uniformly reversible, and accurate differentiation from true disease progression requires integration of serial imaging, biochemical trends, and clinical context. In this patient, sustained biochemical improvement and absence of radiologic progression supported the interpretation of pseudocirrhosis as a treatment-related remodeling process, which did not warrant interruption of effective HER2-targeted therapy.

This report has limitations. Severe hepatic dysfunction remains an exclusion criterion in clinical trials, restricting available evidence to case-based experiences and heterogeneous retrospective cohorts. Furthermore, the extreme biochemical impairment observed here exceeds that described in most published cases, limiting generalizability. Ongoing surveillance is essential to distinguish treatment-related hepatic remodeling from evolving hepatic dysfunction, particularly when pseudocirrhosis features emerge.

In conclusion, this case shows that hepatic visceral crisis in HER2-positive metastatic breast cancer can be reversible even with extreme hepatic dysfunction (bilirubin 31.4 mg/dL). Recovery was driven mainly by uninterrupted dual HER2 blockade, with dose-reduced paclitaxel providing brief initial cytoreduction. Radiologic pseudocirrhosis represented treatment-related remodeling rather than progression, underscoring the need for careful interpretation. This experience highlights the value of a biology-centered approach in HER2-driven visceral crisis and supports expanding trial eligibility to selected patients with severe organ dysfunction who may benefit from targeted therapy.

## Patient perspective

“At the time of admission, I experienced severe fatigue, abdominal discomfort, and progressive jaundice, which significantly affected my daily functioning. I understood that my condition was serious, although I was not fully aware of how critical it was at that moment. The initial phase of treatment was challenging, but I began to notice progressive improvement within the first weeks, particularly with the resolution of jaundice and recovery of energy levels. Over time, my condition improved substantially, allowing me to regain independence in daily activities. This recovery was unexpected given the severity of my initial presentation. I am grateful for the care I received and the treatment approach that was followed. I hope that sharing my experience may be helpful for patients facing similar situations.”

## Data Availability

The original contributions presented in the study are included in the article/supplementary material. Further inquiries can be directed to the corresponding author.
